# Visual Features in Alzheimer's Disease: From Basic Mechanisms to Clinical Overview

**DOI:** 10.1155/2018/2941783

**Published:** 2018-10-14

**Authors:** María Alejandra Cerquera-Jaramillo, Mauricio O. Nava-Mesa, Rodrigo E. González-Reyes, Carlos Tellez-Conti, Alejandra de-la-Torre

**Affiliations:** ^1^Escuela Superior de Oftalmología-Instituto Barraquer de América, Bogotá, Colombia; ^2^Grupo de Investigación en Neurociencias (NeURos), Escuela de Medicina y Ciencias de la Salud, Universidad del Rosario, Bogotá, Colombia

## Abstract

Alzheimer's disease (AD) is the leading cause of dementia worldwide. It compromises patients' daily activities owing to progressive cognitive deterioration, which has elevated direct and indirect costs. Although AD has several risk factors, aging is considered the most important. Unfortunately, clinical diagnosis is usually performed at an advanced disease stage when dementia is established, making implementation of successful therapeutic interventions difficult. Current biomarkers tend to be expensive, insufficient, or invasive, raising the need for novel, improved tools aimed at early disease detection. AD is characterized by brain atrophy due to neuronal and synaptic loss, extracellular amyloid plaques composed of amyloid-beta peptide (A*β*), and neurofibrillary tangles of hyperphosphorylated tau protein. The visual system and central nervous system share many functional components. Thus, it is plausible that damage induced by A*β*, tau, and neuroinflammation may be observed in visual components such as the retina, even at an early disease stage. This underscores the importance of implementing ophthalmological examinations, less invasive and expensive than other biomarkers, as useful measures to assess disease progression and severity in individuals with or at risk of AD. Here, we review functional and morphological changes of the retina and visual pathway in AD from pathophysiological and clinical perspectives.

## 1. Introduction

Alzheimer's disease (AD) is the most prevalent progressive neurodegenerative disease in humans and the main cause of dementia worldwide [1]. Together with vascular dementia, AD corresponds to 60%–80% of all dementia cases [2]. According to the World Alzheimer Report [3], an estimated 47 million people worldwide currently have dementia, a number that is projected to rise to approximately 131 million by 2050. Several modifiable and nonmodifiable risk factors have been described for AD, although aging remains the most significant [4]. Consequently, after 65 years of age, AD prevalence is expected to double every 5 years [5]. According to the US National Institute on Aging-Alzheimer's Association workgroup, there are three different stages of AD: preclinical AD (presymptomatic with early AD-related brain changes observed by neuroimaging or other biomarker studies such as amyloid and tau studies in cerebrospinal fluid (CSF)), mild cognitive impairment (MCI) caused by AD (mild cognitive decline but still able to perform daily activities), and dementia caused by AD (major cognitive decline that interferes with daily activities) [6]. Globally, AD has significant direct and indirect costs, which increased from US$ 604 billion in 2010 to US$ 818 billion in 2015, totaling around 1% of the worldwide gross domestic product (GDP) [3].

Two types of AD have been characterized based on age of onset: early-onset AD (EOAD), referred to as familial, and late-onset AD (LOAD), which is regarded as sporadic. EOAD presents before the age of 65 and corresponds to around 5% of AD cases [7], while LOAD appears after 65 years of age and represents the most common type of AD, accounting for >90% of AD cases [8]. No direct cause has been described for LOAD, but its disease development is influenced by various environmental and genetic risk factors [9]. The main risk factors include aging, genetics (presenilin 1 (PSEN1), PSEN2, and APP1 genes) [7, 10], hypertension, diabetes, hypercholesterolemia, stroke, obesity, sedentary lifestyle, depression, low socioeconomic status, and alcohol and tobacco use [1, 4, 9].

AD is characterized by formation and aggregation of extracellular plaques of abnormal amyloid-beta (A*β*) peptides, as well as presence of intracellular aggregates of hyperphosphorylated tau protein, known as neurofibrillary tangles (NFT) [11, 12]. In AD, A*β* peptides are produced from abnormal cleavage of the amyloid precursor protein (APP) by the beta-site amyloid precursor protein cleaving enzyme 1 (BACE-1 or *β*-secretase) and *γ*-secretase complex [13]. A*β* peptides promote intracellular tau phosphorylation, with hyperphosphorylated tau protein posteriorly aggregating into NFT [11, 14]. A*β* and NFT activate astrocytes and microglia, inducing production of proinflammatory factors such as interleukins and nitric oxide (NO), and ultimately resulting in excessive neuroinflammation, oxidative stress (OS), neuronal damage, and cell death [15–17].

Currently, the diagnosis and classification of AD are based on clinical and neuropsychological examinations complemented by neuroimaging studies. Nonetheless, a conclusive diagnosis still relies on pathological examination of postmortem brain tissue [18]. While many exploratory biomarkers have been proposed for AD, cerebrospinal fluid levels of tau and A*β* levels, together with imaging studies (such as positron emission tomography carbon 11-labeled Pittsburgh compound B), are the most widely accepted [19]. Unfortunately, these techniques are not easily accessible to patients with dementia worldwide owing to elevated costs and a lack of adequate medical facilities. Therefore, it is important to find easy-to-acquire, cost-effective methods that can provide information on the main disease manifestations, even at early stages, to favor timely interventions and improve the quality of life for patients. In this regard, a recent strategic roadmap aimed at improving early diagnosis of AD based on biomarkers was proposed and is centered on several phases that evaluate analytical validity, clinical validity, and clinical utility [20].

The visual system may be a helpful marker for the early stages of AD. Brain alterations in AD can be accompanied by ocular symptoms [21–24] which may be related to progression, cognitive deterioration, and disease severity [25]. This may be explained because the visual system shares the same embryological origin, namely, the neural tube of the brain, and indeed is considered an extension of the diencephalon [26]. In addition, both components have several functional and structural similarities, including microvasculature and neuronal projections [27]. Furthermore, examination of the visual system may provide markers for identifying dementia subtypes, thereby helping to differentiate AD from vascular dementia [28].

Hence, the aim of this review is to describe functional and morphological changes of the eye and visual pathway that are observed in AD from a clinical and pathophysiological perspective. Considering that the visual system shares vascular and cellular components with the central nervous system (CNS), ocular alterations observed in patients with AD may represent an initial manifestation of the disease and serve as possible candidates for complementary diagnostic biomarkers of MCI and the early stage of AD.

## 2. Pathological Mechanisms of A*β* and Tau Toxicity in the Eye

### 2.1. General Mechanisms of A*β* and Tau Production

Senile plaques (SP) are formed from extracellular aggregation of A*β* deposits, which are derived from excessive or inadequate cleavage of APP by BACE-1 and the *γ*-secretase complex [13]. PSEN1 and PSEN2 are part of the *γ*-secretase complex, which also includes nicastrin, anterior pharynx-defective 1, and presenilin enhancer 2 [29]. Although its function is not fully understood, APP has been involved in regulation of synaptic plasticity and acts as a cell adhesion molecule that participates in several neurodevelopmental aspects such as migration, neurite outgrowth, growth cone pathfinding, and synaptogenesis [30, 31]. Inadequate cleavage of APP may lead to formation of abnormally long peptides, such as A*β*1–42 fragments, which are more likely to oligomerize and form plaques, compared with shorter fragments such as A*β*1–40 [32]. Although the precise neurotoxic mechanisms of A*β* are not yet fully elucidated, the presence and aggregation of these peptides compromise the function of various cells including astrocytes, microglia, and neurons, leading to systemic failure of brain activity, mainly related, but not limited, to cognitive aspects [33, 34].

*In vitro* evidence indicates that A*β* induces the release of proinflammatory factors from astrocytes and microglia, including interleukin-1*β* (IL-1*β*), IL-6, NO, and tumor necrosis factor alpha (TNF*α*) [15, 16]. In addition, A*β* oligomers may generate free radicals and induce mitochondrial dysfunction via abnormal activation of glial cells [35, 36]. Other pathological mechanisms include glutamate-induced neuronal excitotoxicity [37], GABAergic dysfunction [38], and reduced cerebral glucose intake [39, 40]. Ultimately, accumulation of A*β* peptides may trigger intracellular signaling for tau hyperphosphorylation, with formation of tau oligomers and NFT [11, 14] leading to neuronal degeneration [41]. Tau appears to be involved in disease progression, as tau aggregates propagate in a prion-like fashion, initiating a self-amplifying cascade and spreading to other brain regions [42]. In AD, these aberrant proteins are deposited in several brain areas including the frontal, parietal, temporal, and occipital lobes and cause neuroinflammation, OS, metabolic dysfunction, excitotoxicity, and perturbation of synaptic plasticity [43–48].

### 2.2. A*β* Mechanisms in the Retina

Retinal cells share a common embryonic origin with the brain and indeed are a projection of the CNS [49]. Further, they have similarities in vasculature, glial cells, neurotransmitter systems, and connectivity with the visual cortex [50]. Moreover, insoluble aggregation of A*β* peptides in the eye of double transgenic AD mice has been observed in different retinal layers, mainly the ganglion cell layer (GCL), nerve fiber layer (NFL), photoreceptor layer, and inner plexiform layer (IPL), leading to neuronal damage and visual symptoms [51]. Although this has not been confirmed in humans, it is hypothesized that similar changes may occur.

Glial cells play a crucial role in the biochemical and molecular mechanisms that induce neurodegeneration and are highly expressed in the mammalian retina, optic nerve, and retinal blood vessels. Astrocytes predominate in the NFL, interacting with retinal ganglion cells and in close relationship with blood vessels of the inner nuclear layer (INL) [52]. Thus, A*β*-induced toxic effects on astrocytes and microglia may generate alterations in the eye and visual system like those found in other nervous tissues, such as the hippocampus or cortex.

Moreover, A*β* has been shown to produce changes in Müller cells, the main glial cells resident in the retina. An *in vitro* model using the immortalized human Müller cell line MIO-M1 reported that treatment with A*β*_1–42_ induced caspase-independent apoptosis through activation of the purinergic receptor P2X7 in these cells [53]. A subretinal injection of A*β*_1–42_ in C57BL/6 mice stimulated the development of gliosis and a proinflammatory phenotype in Müller cells, together with an increase in protein expression of cyclooxygenase 2 (COX-2), glutamine synthetase (GS), the inwardly rectifying potassium channel Kir4.1, and the aquaporin-4 (AQP-4) water channel [54]. However, studies in AD animal models have found contradictory results. A study using the triple transgenic mouse model, 3x-TG-AD [55], observed strong gliotic responses and increased processes in Müller cells close to amyloid deposits, while a more recent study using the APPSWE/PS1ΔE9 mouse model failed to see any significant gliotic response or changes in GS expression [56].

**Figure 1:**
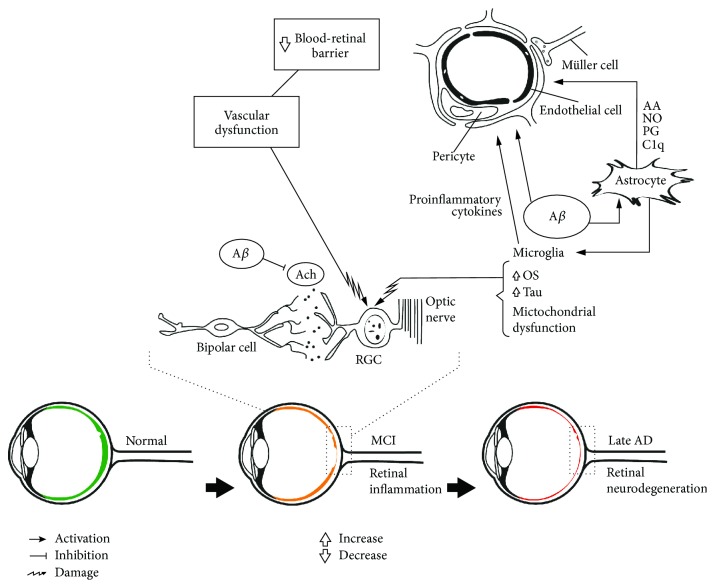
Pathophysiological events in the retina during Alzheimer's disease progression. Amyloid-beta (A*β*) induces microglia and astrocyte activation, synaptic dysfunction, and neurodegeneration. These interactions can be observed by noninvasive ophthalmological examinations in the retina at different stages of Alzheimer's disease (AD). Reduction in the peripapillary region and macular volume has been described in MCI and AD. AA: arachidonic acid; A*β*: amyloid-beta; ACh: acetylcholine; AD: Alzheimer's disease; MCI: mild cognitive impairment; NO: nitric oxide; OS: oxidative stress; PG: prostaglandins; RGC: retinal ganglion cells.

Aggregation of A*β* plaques can lead to inflammatory reactions, reactive gliosis, and cellular and neuronal apoptosis in both the brain and retina [43, 57]. At the very early disease stage, an increase in macular layer size is observed in amnestic MCI patients, which may correspond to inflammatory processes and/or gliosis [58]. Also, astrocytes induce the release of vascular factors such as prostaglandins, NO, and arachidonic acid, which may lead to neurovascular dysfunction in AD as indicated by pathological and experimental studies [59–61]. Similarly, it has been shown that levels of complement (C1q) are elevated in close vicinity to amyloid plaques and astrocyte processes in transgenic mice [62]. In addition, C1q activation generates endothelial cell adhesion in human dermal microvascular endothelial cells [63]. Together, these mechanisms may affect retinal vessels and metabolic supply and increase metabolic injury in the GCL of the retina in the early stage of AD as well as during MCI (Figure 1).

Several studies in humans and animal models have identified A*β* plaques in the photoreceptor layer, GCL, NFL, IPL, and outer plexiform layer (OPL), which manifest at the early stage of disease, even before the appearance of neurological symptoms [64–66]. The retinal and visual effects of these plaques are not entirely clear, but activation of astrocytes and microglia, together with neuroinflammatory changes induced by A*β*, lead to an initial increase in the retinal epithelium (RPE) and GCL degeneration in a transgenic rodent model of AD [64, 67]. In addition, an increase in the number of astrocytes and astrocyte/neuron ratio (which is associated with increased neuronal loss), especially in the superior and inferior quadrants of the retina, has been reported in humans [68]. Similar changes where neuronal numbers are reduced and nonneuronal cells are increased have been observed in human brain regions such as the hippocampus and cerebral cortex [69], as well as in the visual cortex [70]. Nonetheless, despite the previous observations of A*β* effects on the eye, there are limitations to mimic the clinical findings in animal models, which may point to other endogenous mechanisms present in these models and diverse adaptive changes in early phases of AD [71, 72]. Additionally, it is noteworthy to remark that A*β* aggregation was also shown within the eye of individuals undergoing normal cognitive aging; however, this pathophysiological process is quite distinct from the one described in patients suffering AD as it is discussed below in Section 2.4.

### 2.3. Tau Mechanisms in the Retina

One of the hallmarks of AD is the presence of intraneuronal hyperphosphorylated tau protein. These tau oligomers assemble in large insoluble fibrillar structures, known as NFT, and spread between cells in both the brain and retina. Tau oligomers seem to be one of the main structures that promote inflammation in the retina at the early disease stage. Tau oligomers induce neuroinflammation in a perpetuating feed-forward cycle, propagating neural damage, augmenting tau oligomer production, and consequently triggering the appearance of more inflammation [73]. Other studies detected tau accumulation in the retina at an early phase of AD: before the onset of cognitive impairment and even before tau aggregation in the brain in the mouse model of AD. In the retina, tau accumulates principally in GCL dendrites and intraretinal axons, but is depleted in optic nerve axons [74]. Some studies have demonstrated predominant accumulation of tau in GCL, but it has also been observed in the INL, IPL, OPL, and NFL [65, 75]. Conversely, hyperphosphorylated tau has not been found in other structures such as optic nerve, cornea, and lens in AD transgenic mice [76, 77]. Tau accumulation causes dysfunction in anterograde axonal transport, which is a feature of neuronal impairment that occurs before the appearance of retinal cell death [74]. Tau aggregation inhibits mitochondrial transport towards the GCL periphery, leading to loss of energy production and formation of reactive oxygen species (ROS). Altogether, retinal neurons in AD tend to be more susceptible to OS, produce hyperphosphorylated tau aggregates, and prolong the delay in vesicular transport that is necessary for synapse function and cellular growth [75]. Future studies comparing tau aggregation between AD patients, MCI, and individuals undergoing healthy aging could be useful to clarify if tau plays any role in retinal changes in AD pathology.

### 2.4. A*β*, Aging, and Age-Related Macular Degeneration

During aging, the brain and retina develop extracellular deposits recognized as plaques and drusen, respectively. “Drusen” in the retina are deposits of insoluble cellular debris and lipids localized beneath the RPE and inner collagenous layer of Bruch's membrane (BM) [71]. Two types of “drusen” have been identified (hard and soft which differ in their location, and in their clinical and morphological appearance). Both soft and hard “drusen” have similar compositions; however, some authors suggest that each type of “drusen” may be composed by a certain variety of proteins or may be constituted by different amounts of the same components. Differences in “drusen's” morphology, composition, physical properties, and location (peripheral or central) could most likely be based on different formative mechanisms that may contribute to macular susceptibility for age-related macular degeneration (AMD) progression. Although “hard drusen” can appear everywhere in the retina, they are usually located in the peripheral retina and are characterized by hemispherical structures with well-defined borders. “Hard drusen” are considered part of the normal aging process. “Soft drusen” are located exclusively in the macula, characterized by poorly defined borders and larger size [78]. They are linked to the early stage of AMD [72, 79]. AMD is a slow, progressive disease that compromises the macula and the primary cause of irreversible blindness during aging [43].

Two types of AMD have been described: dry AMD type, characterized by significant atrophy of RPE and the photoreceptor layer, and wet, or exudative, AMD type, with invasive blood vessels through the blood–retinal barrier [43]. These invasive vessels come from the choroid and proliferate in the retinal layers between BM and RPE or sometimes proliferate between RPE and the neurosensory retina. In turn, this leads to vascular leakage and fluid accumulation, with formation of fibrovascular disciform scars in the retina that can be observed by ophthalmoscopy [79].

The extracellular deposits in the brain and retina share the presence of A*β*, ApoE, proteoglycans, immunoglobulins, metal ions, acute-phase reactants, fibrinogen, prothrombin, membrane debris, and protease elements [43, 72]. A*β* formation is found in both AD and AMD, suggesting a common mechanism related to proteinopathic A*β*1–42 peptide deposition [80, 81] and imbalance between increased A*β* synthesis and reduced A*β* removal [12]. Specifically, in the aging eye, A*β* accumulation occurs primarily in the outer segments of photoreceptors and at the interface between RPE and BM [82]. This process appears to initiate at the apical region of the outer segments and progresses through the retinal layers with age, also accumulating in choroidal blood vessels. In drusen, A*β* is localized in vesicular components known as “amyloid vesicles” or “amyloid assemblies” [43, 78] and exhibits several shapes, diameters, and fusion processes. In addition, some drusen are described as a single large vesicle occupying a sizable portion of the drusen, or densely packed with several amyloid vesicles [43]. Amyloid vesicles are formed by amyloid oligomer cores composed of toxic oligomeric A*β*1–42 peptides that are centrally organized in a ring structure [82], in close proximity to the inner collagenous layer of BM [71]. A*β*1–42 interacts with different drusen components, including complement proteins (HF1, C3b/iC3b, and C5), interleukins (IL-1*β*, IL-8, and IL-33), and lipids (cholesterol and phosphatidylcholine) to form mature A*β* fibrils, which compose the shell of amyloid vesicles [43, 82].

Overall, these interactions increase ROS production, which contributes to formation of glycation and lipoxidation end products [79]. Additionally, it is hypothesized that A*β* promotes retinal microglia translocation from the inner retina (normal position) to the subretinal space, inducing changes in RPE cells and leading to secretion of proinflammatory, chemotactic, and proangiogenic molecules [82]. Furthermore, complement proteins appear to play a key role in the pathophysiological mechanisms of AD owing to activation by membrane proteins, lipids, cholesterol, and A*β* deposits. Complement molecules promote inflammatory stimuli [72], disrupt the RPE junctional complex [82], impair trans-epithelial permeability, and induce mitochondrial damage. Moreover, they also promote an increase in angiogenic factors, such as vascular endothelial growth factor (VEGF), and decrease in anti-angiogenic factors, such as pigment epithelium-derived factor [43, 83].

The role of tau in AMD is debated and likely underestimated, as tau has not been found in drusen, but has been reported in OPL, INL, IPL, and NFL of patients with AD. Some studies suggest that tau aggregation in aged retina causes defective transportation of molecules, cells, and proteins, which may promote drusen formation and subsequently AMD [75].

### 2.5. Apolipoprotein E Mechanisms

ApoE is involved in the transport of cholesterol and triglycerides and is considered one of the most important risk factors for development of AD [84]. ApoE protein is mainly expressed in the brain on astrocytes, but also in microglia, neurons, pericytes, smooth muscle cells, and brain endothelial cells [85]. Of the different ApoE alleles (*ε*2, *ε*3, and *ε*4), ApoE4 shows a strong relationship with increased risk of developing AD, while ApoE2 provides protection against AD [86]. ApoE4 interacts with A*β* to increase A*β* aggregation [87]. Furthermore, the risk of amyloidosis in AD is suggested to be related to the interaction between ApoE, aging, and amyloid burden [88].

ApoE4 is associated with vascular pathology in AD and promotes cerebral amyloid angiopathy. Further, ApoE4 is related to other vascular diseases such as atherosclerosis, stroke, cerebrovascular disease, and coronary heart disease. Consequently, it has been proposed that the pathological effects of ApoE4 are initiated by cerebrovascular insult, which subsequently leads to neurodegeneration. In the retina of transgenic mice, ApoE4 is implicated in reduced vascular density and increased vascular buds and branching in early postnatal days, which ends during the neonatal period. These findings are associated with decreased synaptic expression after the neonatal period, due to low VEGF levels in ApoE4 models. VEGF is implicated in angiogenesis and vascular plasticity and as a neuronal growth factor in retina development. This determined that ApoE4 has transitory synaptic and vascular effects during retinal development, before and after the neonatal period, respectively. Moreover, VEGF in aged retina has proangiogenic effects that promote macular edema, diabetic retinopathy, and macular holes [89, 90].

ApoE can be expressed in RPE, GCL, and BM and is synthesized in Müller cells [89]. ApoE is involved in uptake, processing, transport, and clearance of lipids for retinal metabolism; therefore, disturbance of this process may lead to cholesterol and lipid accumulation in BM [43]. Distinct to AD, ApoE4 confers protection against AMD because it improves BM permeability, facilitates lipid transportation, and consequently leads to reduced debris accumulation in drusen formation. In contrast, ApoE2 increases the risk of AMD apparition because it induces VEGF as a proangiogenic agent [43, 89, 91, 92]. Contradictorily, some studies have shown increased AMD in AD patients [93], while others show no association [94].

## 3. Functional and Pathological Findings in the Visual System

Neuronal and synaptic dysfunction in AD may compromise visual system pathways in associated cortical and subcortical brain structures, as well as peripheral nerves. Complex visual disruptions are described in AD, which are related to neural dysfunction and modifications in varied eye structures. Depending on the area affected, these lead to diverse clinical manifestations, making the visual system a potential diagnostic aid [95].

### 3.1. Cortical, Subcortical, and Brainstem Regions

A*β* and NFT aggregation as well as neurodegeneration and axonal damage are found in different brain structures related to visual system functioning [50]. For instance, in the primary cortex (Brodmann areas 17 and 18) of AD patients, has been described a decrease in neuronal density, and increase in glial density only in area 17. These cellular changes are related with the presence of A*β*-plaques and NFT [70]. In the case of the secondary visual cortex, involvement of the cuneal and lingual areas of the occipital lobe of AD cases may correspond with impairment in selective visual attention, emotion, and visual processing [96–98]. Meanwhile, reduced glucose metabolism in the visual association cortex and inferior parietal cortex might be related to abnormalities in figure copying, color vision tested by isochromatic plates, and stereopsis [99].

Regarding subcortical structures, A*β* aggregation and neuritic plaques have been found in the pulvinar nucleus of the thalamus of AD postmortem samples. This nucleus participates in visual attention and in the control of eye movements [100]. Similarly, amyloid pathology in the lateral geniculate nucleus of the thalamus, the initial processor of visual information, has been reported. In this regard, a reduction in the number of parvocellular neurons and magnocellular gliosis was found [101]. Indeed, the magnocellular system is involved in recognition of movement, spatial perception, and luminosity, meanwhile parvocellular on identification of color and form. Correspondingly, color and motion perception is affected in AD patients [21]. Another structure involved would be the suprachiasmatic nucleus, responsible of the circadian cycle, sleep, melatonin secretion, pupil regulation, and learning. In fact, clinical and animal models of AD indicate severe disturbance in the circadian rhythms, as well as neurodegeneration in brain structures which control those cycles [102–105]. Altogether, cortical and subcortical dysfunction of the visual system may partially explain the difficulties AD patients have with writing, reading, color and structure differentiation, facial recognition, and visual acuity.

Amyloid aggregation, NFT, and neuritic plaques in the superior colliculus, responsible for the onset of ocular movements, have been reported in postmortem samples from the brainstem of AD patients [106, 107]. Accumulation of A*β* has also been observed in the Edinger-Westphal nucleus of the parasympathetic oculomotor system (which is responsible for pupillary constriction), resulting in degeneration, neuronal loss, and decreased acetylcholine (ACh) in this nucleus, with consequent pupillary reflex alterations [22, 46]. The sympathetic system, involved in the dilatation of the pupil, is also affected in more advanced AD stages [46].

ACh is well known as an excitatory neurotransmitter in the learning and memory process [108] and is compromised in AD. Indeed, changes in neurotransmitter function may explain retinal and visual dysfunction in early AD and MCI before neurodegeneration is established. Different studies have shown neurotransmitter synthesis in amacrine cells, and nicotinic receptors in bipolar cells, horizontal cells, photoreceptors, and the GCL [108, 109]. In the retina of a transgenic mouse model of AD (Tg-SwDI), specific cholinergic cell loss together with reactive gliosis was found [108]. Cholinergic dysfunction induced by A*β* is established at distinct levels: neurodegeneration of cholinergic cells [110], ACh depletion [111], impaired ACh release [112], and finally A*β*-induced impairment of muscarinic [113] and nicotinic acetylcholine receptors (e.g., *α*7-nAChR) [114], as well as associated effectors (e.g., potassium voltage-gated channels, KCNQ) [115–117]. In older animal models, downregulation of *α*4, *α*7, *α*9, and *α*10 nAChR, and m4 and m5 muscarinic acetylcholine receptor (mAChR) subunits, was found. These changes are observed in photoreceptors and INL cells, accompanied by cholinergic cell loss in the retina [108]. Contrast sensitivity and visual attention has been found to be deteriorated in AD patients [118]. In this regard, the use of cholinergic drugs such as donepezil leads to improvement in visual selective attention [119]. Similarly, donepezil also increases contrast sensitivity and accuracy in non-AD subjects [120]. Therefore, the stimulation of the cholinergic system in AD patients not only may generate improvement in learning and memory processes but also might improve visual alteration at retinal, subcortical, and cortical levels.

### 3.2. Retina

In the retina, GCL, NFL, IPL, and the photoreceptor layer can generate A*β*, as each layer expresses APP [30]. As described earlier, aberrant APP cleavage leads to neurotoxic A*β* aggregation, enabling inflammatory processing, degeneration, and thinning in all quadrants of the retina in these layers, but mainly in the superior and inferior quadrants. These layers are present in a third of the macula; therefore, any alterations can contribute to reduced macula and fovea volume, which may partially explain the ocular manifestations that these patients exhibit [25, 48, 121–124]. Study in retinas from postmortem patients with AD found a reduction in the number of GCL neurons related to progressive aging [125]. In contrast, GCL thinning was aggravated in patients with AD and not related to aging in other studies [126]. AD ocular findings in human studies are summarized in Table 1, and findings in animal studies are summarized in Table 2.

### 3.3. Optic Nerve

Analysis of the optic disc has been performed in AD patients, with increased cup-to-disc ratio [50], higher pallor area-to-disc area ratio, and decreased disc rim area of the optic nerve observed as indirect signs of axonal damage [21, 127, 128]. These optic nerve alterations are due to degeneration of axons and larger cells of the GCL and NFL [127] in the central and peripheral portions of the retina, which are involved in the magnocellular pathway and ensure greater optic nerve caliber [129]. In addition, reduced hemoglobin percentage in the neuroretinal rim and increased cup-to disc ratio (by 39%–43%) have been demonstrated [128, 130, 131]. Nonetheless, despite significant reduction of the density of axons from the optic nerve in AD when compared with the age-matched control subjects, no correlation between optic nerve volume and cerebral volume in AD patients was observed [132].

### 3.4. Retinal Vasculature

Regarding the vasculature, A*β* protein can be found in the walls of retinal blood vessels. Its integrity is important in maintaining the blood–brain barrier [44]. However, with aging, A*β* protein aggregates and accumulates in blood vessels of the cerebral and retinal vasculature [126]. As already described, retinal microvasculature has the same embryological origin as brain microvasculature, sharing disease-specific pathological mechanisms [44]. Different clinical studies have shown venular narrowing [133] with a decrease in flow, vessel diameter [134, 135], arteriolar–venular fractal dimension [133], and branching pattern [136] in AD. Similarly, increased arterial tortuosity has been observed in clinical studies [133], with standard deviation of vessel width [136] and amplitude of arterial pulsation [126, 133, 136, 137]. Moreover, these findings relate to cognitive impairment, disease progression, and reduction in NFL diameter in the superior quadrant [21, 26, 27, 138–140]. Although other studies differ in these conclusions, no significant variations were shown for arteriolar–venular caliber, branching angle, or venular tortuosity in AD patients [133].

## 4. Visual Impairments in AD: Clinical Features

AD affects different structures of the visual system in a similar pathological manner as the brain, with manifestation of distinct signs and symptoms that can be determined by adequate clinical history and ophthalmological examination. To our knowledge, there are no studies showing the chronological clinical manifestations specifically correlated with changes in the retina in patients with AD. Therefore, it is important to conduct clinical prospective studies in order to clarify this aspect. Regarding structural changes in the retina, thinning of the superior or inferior quadrants has been described in AD patients associated with visual field impairment. Other visual findings have been correlated with alterations in the brain [141]. Regarding early visual manifestations, it has been observed that contrast sensitivity is the first visual dysfunction found in animal models and AD patients [142, 143]. The rest of visual symptoms are evident depending on the compromised brain structure [22, 95, 144].

Complex visual disturbances are described in patients with AD, including constructional and visuoperceptual disorientation, specifically difficulties in searching for objects (figure–ground discrimination), finding their way in familiar surroundings (environmental agnosia) [99, 145], and spatial recognition or spatial order memory (spatial agnosia) [146]. Additionally, distinct alterations in daily life activities due to deterioration in visual attention [147] and impairments in tasks requiring semantic access from visual information (e.g., disproportionate reading [148] and facial recognition [149]) are observed. With facial recognition, transient misidentification, abnormal self-identification, Capgras syndrome (denial of a familiar person and identifying them as a replacement, imposter, or double), and in some cases prosopagnosia (disordered recognition of familiar faces) have been described [150]. These disturbances are due to disordered visual processing because of disrupted corticocortical projections as a result of alterations in cortical and visual pathways (i.e., occipito–parietal and occipito–temporal) [151, 152]. Disordered visual processing can lead to complications in perceiving and comparing visual stimuli, alterations in processing visual stimuli, and impairments in long-term memory and generating verbal or action responses [148].

Another visual disturbance identified in AD is Balint's syndrome. This is an uncommon and incompletely understood visuospatial defect reported in patients with bilateral parieto-occipital damage associated with Brodmann's area 7b in the posterior parietal lobe (which is involved in control of pursuit ocular movements and target gaze on a stimulus) and superior occipital cortex areas 17, 18, and 19 (which are involved in visual field construction) [153]. Balint's syndrome is characterized by simultanagnosia (difficulties in locating, reaching, or attending to multiple items in a visual space), ocular motor apraxia (inability to maintain fixation on a specific point located in the peripheral visual field), and optic ataxia (inappropriate coordination of voluntary movements in response to a visual stimulus) [149].

Recently, alterations in ophthalmologic examination of AD patients have been described. These alterations include decreased visual acuity, with difficulties in writing and reading [154] increasing along with disease progression [139, 155, 156]. Similarly, compromised contrast sensitivity (ability to distinguish an object on a background of different frequencies) [142–144, 157] has been observed as a principal manifestation during the initial disease stage, with progressive impairment throughout the disease course [143]. Contrast sensitivity is associated with damage to the magnocellular pathway in the geniculate nucleus of the CNS, which is involved in the ability to identify luminosity and motion perception [158]. Another manifestation in AD patients is fluctuations in color perception, mainly errors in color recognition, due to involvement of the parvocellular pathway, which is characterized by smaller axons of the optic nerve [159]. Additionally, pupillary reflex deficits are reported as a deficiency of the sympathetic and parasympathetic systems, with the principal findings being decreased amplitude, speed, and latency of reflexes. Further, smaller resting pupil diameter and maximum dilatation velocity in the dark [160] as well as reduced amplitude and 75% recovery time of the light reflex response [22, 160] are also reported. Other abnormalities in ocular movement, decreased movement perception, and stereopsis dysfunction have been observed in patients with AD [46]. These abnormalities include inability to focus on an object (less relative fixation time) [161], irregular convergence angle, difficulty performing saccadic ocular movements [162, 163], poorer heading and speed perception at lower temporal periodicity [24], and inability to identify three-dimensional objects [164]. Finally, loss of the visual field mainly occurs in the inferior quadrant and is related to GCL damage in the superior quadrant of the corresponding retina [96]. Visual field loss is characterized by accumulation of larger SP and greater amounts of NFT in the cuneal gyrus than lingual gyrus of the visual cortex, which is where nerve fiber projections of the GCL in the superior and inferior quadrants terminate, respectively [135].

## 5. Paraclinical Visual Studies in AD

Different noninvasive imaging methods have been developed to study structures of the visual system and visual pathways in AD patients. In this section, we describe findings obtained by optical coherence tomography (OCT) and bioelectrical changes through electrophysiological examinations, specifically electroretinograms (ERG) and visual evoked potentials (VEP).

### 5.1. Optical Coherence Tomography in the Retina

In the retina, different OCT methods based on cross-sectional images of the retinal layers have been performed in AD patients, including spectral domain-optical coherence tomography (SD-OCT). Using SD-OCT, thinning and thickening of NFL in the superior and inferior quadrants, respectively, of the nasal and temporal regions were related [165] to cognitive impairment [131, 166, 167]. Degeneration of NFL, GCL, and IPL has been related to AD duration, with longer disease duration associated with greater damage to the retinal layers. The most affected quadrants were the temporal, temporal–inferior, and temporal–superior sectors, with increased nasal/temporal ratio reflecting higher temporal sector commitment. In addition, reduction of the GCL was associated with axonal degeneration, which may predict greater disease severity [168].

Among other OCT studies, progressive compromise of the NFL was shown between an intermediate group with MCI and different disease stages. Thinning of NFL in the superior quadrant was gradually affected among patients with MCI and mild AD. The inferior quadrant was involved in severe disease stages, demonstrating engagement of the NFL throughout disease progression [169]. In addition, another study detected a decrease in NFL in AD and MCI groups in the inferior quadrant, with the superior quadrant significantly compromised in AD patients only [170]. Contrarily, NFL thinning was evident in all MCI and AD groups, indicating that NFL involvement is observed in the early disease stage [171]. Furthermore, significant NFL thinning was identified in patients at the early stage of AD [165]. A meta-analysis of 51 articles in the last 5 years recognized that loss of NFL mainly occurred in the superior quadrant, yet corresponded to visual field impairments in the inferior quadrant. This deterioration in NFL was related to disease duration in AD [140].

When comparing AD patients and healthy controls, a significant NFL reduction has been observed in AD patients, more prominent in the inferior [172, 173] and superior quadrants [140, 174]. A meta-analysis including 887 AD patients and 864 controls evidenced peripapillary NFL thinning in the superior and inferior quadrants of the retina in AD patients that was not present in controls [175]. Additionally, AD patients had reduced thickness in GCL, NFL, IPL, and outer nuclear layer (ONL) compared with controls [176].

In contrast, a relationship between NFL involvement (through OCT) and cognitive impairment by Mini-Mental State Examination (MMSE) was reported in AD patients, suggesting that measurement of NFL may be used as a disease progression marker [177]. However, other studies have observed NFL thinning in all quadrants, especially the nasal region, with no relationship to the MMSE and AD severity [178].

### 5.2. Optical Coherence Tomography in the Macula

The macula is the principal structure affected at the early stage in AD patients. By OCT, decreased macula volume was observed in the four internal quadrants and one temporal external quadrant, with no significant difference in decrease in peripapillary region volume in AD patients [179]. However, a recent meta-analysis has shown that AD and MCI patients had lower peripapillary retinal NFL compared with controls [180]. Using SD-OCT, increased macular size and IPL was shown in patients with MCI and those at risk of developing AD, suggesting an inflammatory process and reactive gliosis in the early disease stage before establishment of AD [172, 174]. Conversely, macular thinning was identified in MCI [178] and in all sectors except the fovea, mainly in NFL of the superior quadrant in AD [156]. Decreased IPL in the inferonasal, inferotemporal, and superotemporal sectors was observed, as well as reduced NFL in the temporal sector in patients with MCI who developed AD two years later. Corroboratively, these results were related to cognitive impairment [176]. Macular atrophy has been described as the first manifestation in patients with AD and is related to degree of cognitive impairment in the MMSE [140]. By frequency domain optical coherence tomography in AD, NFL, GCL, and IPL are shown to be reduced in size in the macula, except in the inferior external quadrant. Again, this thinning is related to MMSE results [181].

### 5.3. Optical Coherence Tomography in the Choroid

Choroid reduction and thinning are observed in AD. Using SD-OCT, thinning of the choroid was measured in the macula at 13 locations (separated by 500 mm) in elderly compared with healthy subjects. A significant decrease in the choroid was shown in all 13 locations, but principally in two locations temporal to the fovea. This indicates that compromise in the choroid is not related to age [182]. By OCT examination over a period of 12 months, greater choroid reduction was characterized in AD patients compared with controls of the same age [183]. Another study measured thickness of GCL in the macula, the choroid, and external retina, and compared them with MMSE results. GCL thinning in the superior and inferior quadrants of the macula was observed, with significant thinning of the choroid in all regions. Further, a relationship was observed between GCL thinning in the macula and MMSE results, but not with choroid thinning, which was unrelated to severity of cognitive damage [173].

### 5.4. Electrophysiological Examination

Electrophysiological examinations (i.e., bioelectrical changes in the visual system) such as VEP and ERG have been performed in patients with AD [140]. VEP extracts signals directly from the visual cortex, which are dependent on adequate integrity of the visual system (eye, retina, optic nerve, and visual cortex). In patients with AD, decreased amplitude of P2 or P100 components are observed at 100 ms [45, 46, 48, 96, 138–140, 175, 184–186]. Yet, other studies have shown no changes in pattern-VEP nor association with NFL thinning [187] in the peripapillary or macular regions [188].

The pattern ERG (PERG) test presents three peaks: negative–positive–negative, which are observed at 35–50–95 ms, respectively [46]. Most studies show decreased amplitudes and time delays, mainly in N35, P50, and N95 components, in PERG of patients with AD [140, 189]. In contrast, only reduced P50–N95 amplitudes with increased implicit P50 time [190] were shown in other studies, while a compromised magnocellular pathway was also reported [158]. All these findings relate to damage at the GCL level (N95) [190]. Conversely, further studies detected no PERG changes [191]. In another type of exam, the multifocal ERG, which evaluates macula functionality, even in early or intermediate states of the disease in patients with AD, a statistically significant reduction in the electric activity of the macula has been registered, especially in the foveal and parafoveal regions, due to the decrease in the amplitude of the P1 component [145]. In PERG, reduced mean amplitudes in these components were observed in patients with AD. This anomaly may be due to axonal depletion within the optic nerve as well as GCL degeneration [192]. Additionally, PERG at different frequencies showed amplitude reductions at high frequencies in patients with AD [193]. In other types of examination, namely, pattern-reversal VEP and ERG, no alterations were detected, but delays were occasionally demonstrated in the second positive component of flash VEP [192]. Scotopic and photopic flash ERG and oscillatory potentials show no alterations between both methods in patients with AD, suggesting no visual pathway involvement [194]. With VEP, decreased latency in the magnocellular pathway was observed in AD patients [158].

## 6. Future Clinical Directions

The clinical study of the visual system offers several benefits owing to the relatively scarce invasiveness of the examination, as well as the associated low cost of procedures. Structural eye components such as the cornea, lens, retina, and optic nerve can be observed with an ophthalmoscope. Additionally, functional aspects related to autonomic features, such as pupillary reflex, can be directly studied. Further, a wide array of visual paraclinical tests have been developed including VEP, ERG, tonometry, and OCT. These procedures are not limited to evaluating aspects of the visual system and can also be used to examine neurological or systemic components of many conditions. This has led to the proposition that studying the visual system is a helpful and complementary approach with biomarker potential in neurodegenerative conditions, including AD [45]. Many visual changes manifest in AD patients (as already discussed), including alterations in visual acuity, color perception, contrast sensitivity, the visual field, pupillary reflexes, ocular movements, and stereopsis. All can be examined by simple, noninvasive, and easily accessible methods such as adequate ophthalmologic examination, OCT, and electrophysiological examinations. These are supporting diagnostic tools that could potentially even be used in the early disease stage. Current biomarkers for AD tend to be either expensive or invasive, and in particular for countries with poor and inadequate healthcare systems. Moreover, no current biomarkers predict with absolute confidence, conversion of MCI to AD, or development of sporadic AD. For a recent review on the advantages and disadvantages of current AD biomarkers, refer to [20]. Several findings in the visual system point to the useful role of eye examinations for determination of AD. One of the challenges of this approach is to differentiate changes related to normal aging from those of pathological origin. To date, three structural components of the eye, the retina, eye vasculature, and lens, have emerged as interesting research areas in AD [45].

Study of the retina and associated structures has shown AD-related changes in preclinical (phase 1) animal exploratory models, thereby providing hints for their utility as biomarkers in humans. Many of these studies have been performed in transgenic humanized rodent models expressing AD characteristics. Accumulation of A*β* in plaques was observed at 12 months in the retina of transgenic APPswe/PS1ΔE9 mice. These plaques localized mainly in the OPL and IPL and increased in number and size with age [64]. Similar results were also observed in transgenic APPswe/PS1ΔE9 rats, with A*β* plaque-like structures present in the retina, together with choroidal thinning [67]. A*β* peptides were observed in the inner and outer segments of photoreceptors, NFL, and GCL, with higher expression in the central retinal region, in the Chilean rodent, *Octodon degus* [65], which has been suggested as a possible natural model of AD [195]. Some studies in transgenic mice have even reported earlier appearance of plaques in the retina than brain [42]. Furthermore, changes in retinal plaques can be individually monitored in real time following glatiramer acetate immunization in mice [66]. Regrettably, these results using similar animal models have not always been consistent, with contradictory findings reported. In a recently published study on APPswe/PS1ΔE9 mice, researchers were unable to demonstrate the presence of amyloid plaques, dystrophic neurites, neuronal loss, macro- or microgliosis, aberrant cell cycle re-entry, OS, tau hyperphosphorylation, or upregulation of proinflammatory cytokines or stress signaling molecules in the retina [56]. Despite promising results obtained in preclinical models, more investigations are needed to address the reasons why AD-related changes are not constant in all animal models and explore the mechanisms inducing cellular and molecular alterations in the retina. Comprehension of these questions is vital if visual components are to be translated from basic science to clinical practice as reliable biomarkers.

Changes in the human retina have also been reported in relation to AD. A recent meta-analysis, which included 25 studies comprising 887 AD patients, 216 MCI patients, and 864 healthy controls, concluded that AD and MCI patients show decreased retinal thickness measured by OCT [180]. Another meta-analysis, which included a lower number of studies and patients, reached a similar conclusion, corroborating the role of OCT and retinal measurement in assessment of AD [123, 196]. Accordingly, retinal measurements in humans may help distinguish between different neurodegenerative conditions. Patients with frontotemporal degeneration have a thinner outer retina, including a thinner ONL. In contrast, AD patients tend to exhibit inner retinal thinning [197]. Despite these encouraging results, in a similar fashion to the animal models, some reports challenge this evidence. In a recent paper, Tau, A*β*, transactive response DNA-binding protein 43, ubiquitin, and *α*-synuclein were examined by immunohistochemistry in postmortem AD patient brains, showing no evidence of inclusions, deposits, or accumulation of other proteins in any part of the ocular globe [198]. As in preclinical experiments, human studies need to be expanded to clarify these controversial aspects of AD-induced changes in the retina and visual system.

Other aspects of the eye and visual system are also reported to show alterations related to AD, although the retina is the component that has been most thoroughly studied. In the lens, some but not all clinical studies show A*β* deposition [199], while others have concluded that cataract grade or lens opacity is unlikely to provide a noninvasive measure of the risk of developing AD dementia [200]. These controversial results do not support the use of lens examination as a biomarker of AD, underscoring the importance of conducting more detailed, controlled clinical studies with a larger number of patients. Ocular vascular changes have also been reported in AD patients [26, 201], which suggest similar microvascular damage in the eye as the well-described cerebral amyloid angiopathy owing to vascular A*β* deposition [202]. Direct and minimally invasive observation of eye vasculature, mainly retinal, provides an advantage to using vascular changes as a marker of AD. Unfortunately, the current evidence is not strong enough to offer a definitive answer on using this approach as a biomarker for AD. A recent systematic review, which included all published studies applying fundus camera imaging to examine association between retinal vasculature/retinopathy and any form of dementia, found many inconsistent results regarding changes in vessel caliber, tortuosity, and fractal dimension, although the authors concluded that retinopathy was more prevalent in dementia [203]. One possible explanation for the variation in results was suggested to be the absence of histopathological confirmation of AD diagnosis as well as lack of dementia severity reports. However, some studies using immunohistochemistry have reported absence of markers for AD in the visual system (Tables 1 and 2). A recent investigation done on postmortem eye tissue obtained from AD patients failed to report any indication of tau, A*β*, TDP-43, ubiquitin, or *α*-synuclein and showed no evidence of inclusions, deposits, or other protein accumulation in any region of the globe [198]. A similar result was obtained in another postmortem study, where no A*β* deposits or abnormal tau accumulations were detected in the lens, retina, or other structures in the eyes of AD patients [204]. Another study found hyperphosphorylated tau but not A*β* aggregates in postmortem retinas from AD patients [77]. These results contrast previously mentioned studies where AD pathological changes in the eyes were found. Differences in experimental procedures or in AD disease stage may partly explain some of these discrepancies. Nonetheless, the reported absence of AD markers in the eye may suggest that the disease compromises or develops in a dissimilar manner in the brain and in the eye. As well, other biochemical, structural, or imaging markers (i.e., OCT) in the visual system may be more adequate than A*β* or tau in the eye. This points to the need for developing future studies with more precise inclusion/exclusion criteria, larger patient populations with clear AD severity classifications and staging (for example, MCI, EOAD, and LOAD), and standardized measurements for vasculature analysis.

Currently, several clinical trials are being conducted to help clarify the use of visual components as possible biomarkers for AD. The National Institutes of Health- (NIH-) supported webpage that is responsible for the registry of clinical trials (https://clinicaltrials.gov/) has 38 studies related to AD and the eye. These studies have a wide range of objectives including measurement of saccades and eye-tracking, visuospatial attention, retinal thickness, and amyloid deposition. Although studying optic changes in AD is not without controversy, it represents a promising target that needs to be carefully studied in the future, both in preclinical and clinical studies, as the potential benefit of eye examinations as biomarkers for AD, in particular for detection of early changes, could have a significant impact in clinical practice.

## 7. Conclusions

The visual system might rise to be an important biomarker of AD because the functional and pathological mechanisms with the CNS are similar. AD patients manifest a reduction in thickness of the retina, explained by a selective neurodegenerative process induced by A*β*. Although the precise reason why certain retinal layers or quadrants are affected is not clear, neuronal loss is the result of the presence of several mechanisms such as inflammation, oxidative stress, and vascular dysfunction. There are divergent points of view about the relationship between ocular manifestations that need to be clarified. However, a better knowledge of pathophysiological mechanisms could lead to the development of new biomarkers during early stages of the disease. Ocular and visual examinations in AD would be a less invasive and more cost-effective method, than the techniques that are currently used. Noninvasive ophthalmological assessment could complement the AD diagnosis and would be a helpful tool to evaluate the disease progression. Novel functional studies of the visual system, as well as objective measurements of vascular and inflammatory changes in the eye, may play an important role in the evaluation of early stages of AD, including MCI and people at risk of dementia.

## Figures and Tables

**Table 1:** AD ocular findings in human studies.

Ocular histopathological hallmarks in human studies				
Ref.	Population (mean age, range years)	Tissue analyzed	AD stage (early vs. late-onset)	Findings
Williams et al. 2017 [198]	AD *n* = 17 (77) Controls *n* = 2 (77)	Retina, lens, and optic nerve	Late	No presence of tau, A*β*, ubiquitin, TDP-43, or a-synuclein.
Tsai et al. 2014 [67]	AD *n* = 10 (63–95) Control *n* = 10 (63–95)	Nasal and temporal regions of the retina	NS	A*β* plaques in retina and reduced choroidal thickness.
Ho et al. 2014 [204]	AD *n* = 11 (82/61–92) Controls *n* = 6 (74/66–86)	Peripheral nasal retina, posterior retina including the macular region and optic nerve head, and peripheral temporal retina	Late Definite AD (*n* = 9) Probable AD (*n* = 2)	Cytoplasmic *α*-synuclein positivity, but not A*β* deposits and abnormal tau accumulation.
Schön et al. 2012 [77]	AD *n* = 6 (37–79) Controls *n* = 4 (53–60)	Retina	Early and late	Hyperphosphorylated tau but no A*β* plaques or fibrillar tau aggregates.
Koronyo-Hamaoui et al. 2011 [66]	AD *n* = 8 (80/48–94) *n* = 5 (79/65–92) Controls *n* = 5 (76/66–85)	Retina	Late (*n* = 9) Early (*n* = 1)	A*β*40 plaques.
Syed et al. 2005 [205]	AD *n* = 12 (81.9 ± 6.54/69–84) Controls *n* = 13 (78.5 ± 8.57/66–82)	Optic nerve	Late	The density of axons was reduced in both the center and peripheral portions of the optic nerve with preferential loss of the smaller-sized axons.
Blanks et al. 1996 [68]	(GFAP-ir) AD *n* = 12 (64–88) Controls *n* = 19 (63–89) Neuronal and cell numbers (GCL) AD *n* = 11 (16 retinas 45–92 years) Control *n* = 9 (11 retinas 60–89 years)	Retina	Late	Extensive neuronal loss in the entire retina (36.4%), most pronounced in the superior and inferior quadrants, throughout the midperipheral regions (40–49%), and in the far peripheral inferior retina (50–59%). Increase in the astrocyte : neuron ratio. Also, more extensive labeling of glial fibrillary acidic protein immunoreactivity (GFAP-ir) in astrocytes in the GCL, in the Müller cells, and in radial processes.
Curcio and Drucker [206]	AD *n* = 4 (67–86) Controls *n* = 4 (66–86)	Retina	Late	No evidence of GCL loss between AD and controls.
Blanks et al. 1991 [207]	AD *n* = 11 (13 retinas 83/69–93 years) Controls *n* = 11 (14 retinas 78/60–98 years)	Retina	Late	The fovea shows a loss of neurons within the GCL, mainly in large and small ganglion cells.
Sadun and Bassi 1990 [129]	AD (10 optic nerves, 3 retinas 76–89 years) Controls (8 optic nerves, 2 retinas 70–79 years)	Optic nerve and retina	Late	Predominant loss of the largest class of retinal ganglion cells (M cells). Retina of 1/3 AD patients also showed degeneration of GCL and their axons in the NFL.
Blanks et al. 1989 [208]	AD *n* = 16 (76–93) Controls *n* = 19 (60–91)	Optic nerve and retina	Late	Degeneration in the GCL is characterized by a vacuolated appearance of the cytoplasm. Absence of neurofibrillary tangles, neuritic plaques, or amyloid angiopathy.
Hinton et al. 1986 [128]	AD *n* = 10 (10 optic nerves, 4 retinas 73–89 years) Controls *n* = 10 (73–89)	Optic nerve and retina	Late	Widespread axonal degeneration in optic nerves, decreased in the number of cells in GCL, and reduction in the thickness of NFL. There was no retinal neurofibrillary degeneration or amyloid angiopathy.

**Table 2:** AD ocular findings in animal studies.

Ocular hallmarks in experimental animal models		
Ref.	Model	Findings
Oliveira-Souza et al. 2017 [108]	Tg-SwDI mice 6.5 to 15 months	Upregulation of AChR gene expression and significant cell loss in the photoreceptor layer and inner retina on young groups. Specific cholinergic cell loss and increased astrocytic gliosis in the middle-aged and AChR downregulation in older adult groups.
Joly et al. 2017 [209]	Tg APPswe/PS1DE9 mice 3 to 13 months	No A*β* or amyloid plaques were detected in the Tg retinas. However, the CTFb/CTFa ratio was significantly lower. Response mediated by cones was preserved. Retinal-specific processing of amyloid may confer protection against AD and selectively preserve cone-dependent vision during aging.
Nilson et al. 2017 [73]	P301L mouse Htau mouse	Tau oligomers colocalize with astrocytes, microglia, HMGB1, and inflammatory cells in the retina.
Chiasseu et al. 2017 [74]	3xTg mice 3 to 6 months	Age-related increase in endogenous retinal tau accumulation, previous to the reported onset of behavioral deficits, and tau aggregation in the brain. Tau build-up occurred in GCL soma and dendrites, but not in axons.
Chidlow et al. 2017 [56]	Tg APPSWE/PS1ΔE9 mouse 3 to 12 months	No presence of amyloid plaques, dystrophic neurites, neuronal loss, macro- or microgliosis, OS, tau hyperphosphorylation, or upregulations of proinflammatory cytokines in the retina.
Gupta et al. 2016 [47]	Tg APP/PS1 mice 13 to 16 months	Increased A*β* deposition in the retinas with thinning in inner retinal layers and decline in scotopic threshold response. Reduction of axonal density in the optic nerve.
Du et al. 2015 [65]	*Octodon degus* 6.7–70 months	A*β* deposition in the inner and outer segment of the photoreceptors, NFL, and GCL. A*β* expression was higher in the central retinal region than in the retinal periphery. Phosphorylated tau was seen more consistently in NFL-GCL regardless of age.
Pogue et al. 2015 [80]	Tg 5xFAD Tg-AD mice 0–5 months	Presence of A*β*42 peptides in the brain and retina, accompanied by inflammatory markers such as cyclooxygenase-2 and C-protein reactive.
Maharshak et al. 2016 [90]	ApoE3 and ApoE4 targeted replacement mice 4–7–12–120 days old	Transient changes in vascular branching and decrease in retinal synaptic density in the apoE4 mice. Additionally, lower levels of retinal VEGF were observed in apoE4 mice compared to the ApoE3 mouse retinas.
Tsai et al. 2014 [67]	TgF344-AD rat 14–19 months	Reduction in choroidal thickness, hypertrophic retinal pigment epithelial cells, inflammatory cells, A*β* plaques, and upregulation of complement factor C3 in the retina.
Williams et al. 2013 [210]	Tg 2576 mice 14 months	No significant changes in GCL synaptic densities but a highly significant change in mitochondrial morphology. GCL dendritic atrophy preceded cell loss, and this may be due to the accumulations of A*β*.
Zhao et al. 2013 [76]	Tg APP/PS1 mice	Hyperexpression of phosphorylated tau was detected in retina, accompanied with an increase in senile plaques and NFTs. The increased tau phosphorylation was associated with a significant augment in the production of p35 and p25, and upregulation of calpain.
Schon et al. 2012 [77]	Tg P301S mice	*In vivo* detection of fibrillar tau in the retina and the progression of tau pathology over several months was demonstrated.
Koronyo-Hamaoui et al. 2011 [66]	Tg APPSWE/PS1ΔE9 mouse 7–17 months	Retinal A*β* plaques were detected following systemic administration of curcumin in Tg, previous to the appearance of A*β* plaques in the brain.
Perez et al. 2009 [64]	Tg APPSWE/PS1DeltaE9 mouse 12–19 months	A*β* plaques appeared in the OPL and IPL of the retina, displaying syntaxin 1 and ChAT, but no neuronal degeneration was observed. ERG revealed reduction in the amplitudes of a and b waves.

## Abbreviations

ACh: Acetylcholine
AD: Alzheimer's disease
A*β*: Amyloid-beta
AMD: Age-related macular degeneration
ApoE: Apolipoprotein E
APP: Amyloid precursor protein
BACE-1: Beta-site amyloid precursor protein cleaving enzyme 1
BM: Bruch's membrane
CNS: Central nervous system
EOAD: Early-onset Alzheimer's disease
ERG: Electroretinogram
GCL: Ganglion cell layer
IL-1*β*: Interleukin-1*β*
IL-6: Interleukin-6
IL-8: Interleukin-8
IL-33: Interleukin-33
INL: Inner nuclear layer
IPL: Inner plexiform layer
LOAD: Late-onset AD
mAChR: Muscarinic acetylcholine receptor
MCI: Mild cognitive impairment
MMSE: Mini-Mental State Examination
nAChR: Nicotinic acetylcholine receptor
NIH: National Institutes of Health
NFL: Nerve fiber layer
NFT: Neurofibrillary tangles
NO: Nitric oxide
OCT: Optical coherence tomography
ONL: Outer nuclear layer
OPL: Outer plexiform layer
OS: Oxidative stress
ROS: Reactive oxygen species
RPE: Retinal pigment epithelium
SD-OCT: Spectral domain-optical coherence tomography
SP: Senile plaques
VEP: Visual evoked potential.
